# Early Rehabilitation in Children After Ischemic Stroke—Importance and Effects: A Scoping Review

**DOI:** 10.3390/children13070866

**Published:** 2026-06-29

**Authors:** Kamila Perliceusz, Alicja Kowalczyk, Zbigniew Dobrzański, Wojciech Witkiewicz

**Affiliations:** 1 Institute of Social Studies, Faculty of Applied Studies, DSW Ideis University, Strzegomska Street 55, 53-611 Wrocław, Poland; 2 Faculty of Biology and Animal Husbandry, Wrocław University of Environmental and Life Sciences, Norwida 25, 50-375 Wrocław, Poland; 3 Research and Development Centre, Voivodeship Specialist Hospital in Wrocław, 51-124 Wrocław, Poland

**Keywords:** paediatric stroke, early intervention, neurorehabilitation, neuroplasticity, functional outcomes

## Abstract

**Background:** Early rehabilitation after pediatric ischemic stroke may support neuroplasticity and improve long-term functional outcomes. However, rehabilitation practices remain heterogeneous, and evidence-based recommendations regarding the optimal timing and intensity of intervention are limited. **Objectives:** This scoping review aimed to evaluate the available evidence regarding early rehabilitation after pediatric ischemic stroke, identify prognostic factors associated with functional recovery, summarize current therapeutic approaches, and highlight gaps in the existing literature. **Eligibility Criteria:** Eligible studies included children and adolescents aged 0–18 years diagnosed with ischemic stroke and receiving rehabilitation or therapeutic intervention. Studies addressing the timing, intensity, and effects of physiotherapy, occupational therapy, speech and language therapy, neuropsychological intervention, neuromodulation, or multidisciplinary rehabilitation were considered for inclusion. **Sources of Evidence:** A structured literature search was conducted in PubMed/MEDLINE, Scopus, Web of Science, the Cochrane Library, and Google Scholar for studies published between 2000 and January 2025. **Charting Methods:** Data were extracted using a standardized charting form and synthesized narratively because of substantial heterogeneity in study design, populations, interventions, and outcome measures. **Results:** Twenty-one sources met the inclusion criteria. Direct evidence specifically addressing early rehabilitation after pediatric ischemic stroke was limited and consisted primarily of observational studies. A substantial proportion of the available evidence was indirect, originating from studies of perinatal stroke, unilateral brain injury, cerebral palsy, and related pediatric neurorehabilitation populations, as well as clinical guidelines and expert consensus documents. The available evidence suggests potential benefits across motor, cognitive, communication, and functional domains, although the strength and directness of evidence varied substantially. Several studies identified the early post-stroke period as a potentially important window for neuroplasticity, while family involvement, individualized treatment planning, and interdisciplinary care were consistently highlighted as important components of rehabilitation. Evidence supporting neuromodulation techniques remained preliminary and was largely limited to selected pediatric populations. **Conclusions:** The available evidence, although heterogeneous and largely indirect, suggests that early coordinated and multidisciplinary rehabilitation may be beneficial in pediatric ischemic stroke care. However, the current evidence base remains limited, and high-quality prospective studies are needed to establish standardized rehabilitation protocols and determine the optimal timing and intensity of therapeutic interventions.

## 1. Introduction

This research paper addresses the issue of early therapeutic intervention after ischemic stroke in children, analysing its importance, scope, and documented effects in the context of contemporary clinical and neurorehabilitation research. The study draws on a review of the literature spanning pediatric neurology, physical therapy, speech therapy, occupational therapy, and neuropsychology. This scoping review examines how the rapid implementation of integrated therapeutic interventions influences brain plasticity, the course of recovery, and long-term functional outcomes in children. The analysis is based on three complementary theoretical perspectives: developmental neuroplasticity, early interdisciplinary rehabilitation, and family support models in the community.

However, clinical practice remains heterogeneous and lacks standardised protocols. To address these challenges, the research document highlights the need to develop consistent clinical guidelines. Ultimately, the paper advocates for an approach that combines scientific rigour with practical accessibility, enabling optimal rehabilitation and improved quality of life for children after ischemic stroke.

Ischemic stroke in developmental age, although rare compared to the adult population, is one of the most serious acute neurological disorders in children. Its consequences can affect subsequent development, education, social relationships, and the quality of life of the entire family.

However, the literature emphasizes that access to early pediatric rehabilitation is uneven and clinical practices vary between centers, both in terms of the time of initiation of therapy and its scope, intensity, and terminology used. The lack of consistent definitions and standards of practice complicates the comparison of research findings and the formulation of clear recommendations. Therefore, analysis of the population of children aged 0–18 years after AIS is a key starting point for evaluating the effectiveness of early therapeutic interventions and identifying factors that can modify their effects.

This paper adds to existing knowledge by integrating data on early therapeutic interventions specifically for pediatric AIS, providing an operational definition of ‘early intervention’ and comparing standard and emerging therapies. It also highlights research gaps, including the lack of randomized controlled trials, and emphasizes practical recommendations for clinical implementation.

The present review synthesizes evidence from multiple domains of pediatric rehabilitation, including physical therapy, occupational therapy, speech therapy, and neuropsychology, to provide a comprehensive perspective on early intervention after pediatric ischemic stroke. It also proposes an operational classification of the timing of therapeutic initiation—ultra-early, early, and subacute early—adapted from adult stroke rehabilitation models and refined in accordance with available pediatric stroke literature and author consensus, as there is currently no universally accepted pediatric-specific framework. This classification may facilitate clearer interpretation of the research findings and clinical communication. Additionally, the article compares standard rehabilitation approaches with emerging therapeutic strategies, highlighting their potential roles in optimizing functional recovery in children after stroke.

Stroke in adults is more common and has well-established diagnostic and rehabilitation protocols [1,2]. Pediatric strokes, in contrast, differ in pathophysiology, clinical course, and rehabilitation needs, highlighting the importance of early and age-appropriate interventions [1,3]. Recent pediatric stroke guidelines and consensus statements emphasize the importance of age-specific diagnostic pathways, multidisciplinary management, and long-term neurodevelopmental follow-up, highlighting the need for rehabilitation strategies tailored to the developing brain.

The incidence of ischemic stroke in children is substantially lower than in adults, though the interpretation of epidemiological data is complicated by methodological inconsistencies across studies, including variable age ranges, reliance on retrospective designs, and case identification via diagnostic code searches rather than standardized clinical criteria. Perinatal arterial ischemic stroke, defined as stroke occurring between 28 weeks of gestation and 28 days of life, has the highest incidence, estimated at 1/3500 live births. Childhood arterial ischemic stroke, affecting children aged 1 month to 18 years, occurs at a considerably lower rate of 1–2/100,000 children per year, with secondary peaks observed in children under the age of 5 (0.38/100,000 per year), and in adolescence (0.48–0.6/100,000 per year). Two recent prospective studies conducted in Canada and Germany reported lower overall incidence rates of 0.41/100,000–1.72/100,000 children per year, though the German study excluded the neonatal period, limiting direct comparability [4]. These differences in incidence between age groups reflect distinct underlying pathophysiological mechanisms and underscore the need for age-specific diagnostic and rehabilitation approaches.

## 2. Methods

### 2.1. Type of Review

This scoping review was conducted and reported in accordance with the PRISMA-ScR guidelines. No review protocol was prospectively registered.

A structured literature review with a systematic search strategy was conducted to analyze the importance of the timing of therapeutic intervention and its effects in children after ischemic stroke. Given the limited volume of empirical studies directly examining rehabilitation in the pediatric ischemic stroke population, and the significant methodological heterogeneity of the available primary research, the scope of eligible sources was intentionally broadened to include clinical trials, observational studies, clinical practice guidelines, expert consensus documents, and reports from specialist organizations. This approach is consistent with the exploratory nature of scoping reviews, which aim to map the breadth of available evidence rather than synthesize findings from homogeneous study designs alone. Importantly, the type and origin of each source are clearly documented in the data extraction table, and findings derived from empirical studies are distinguished from those based on expert consensus or guideline recommendations throughout the narrative synthesis.

### 2.2. Search Strategy

A comprehensive literature search was conducted in PubMed/MEDLINE, Scopus, Web of Science, Cochrane Library, and Google Scholar to identify studies related to early therapeutic intervention after pediatric ischemic stroke. The search included publications published between January 2000 and January 2025. Only articles published in English or Polish were considered eligible.

The search strategy combined Medical Subject Headings (MeSH) terms and free-text keywords related to pediatric stroke, rehabilitation, timing of intervention, and functional recovery. Boolean operators (“AND”, “OR”) were applied according to database-specific requirements.

The PubMed search strategy was as follows: (“pediatric stroke” OR “childhood stroke” OR “perinatal stroke”) AND (“ischemic stroke” OR “arterial ischemic stroke”) AND (“early intervention” OR “early rehabilitation” OR “timing of therapy”) AND (“neurorehabilitation” OR physiotherapy OR “occupational therapy” OR “speech therapy”) AND (“functional outcomes” OR recovery OR neuroplasticity).

The search strategy was adapted for Scopus, Web of Science, and the Cochrane Library using equivalent subject headings and keywords. Google Scholar was additionally searched to identify potentially relevant articles not indexed in the primary databases. The final search update was performed on 31 January 2025. For Google Scholar, the first 330 results sorted by relevance were screened. Duplicate records identified across databases were removed prior to title and abstract screening.

### 2.3. Eligibility Criteria

Studies were considered eligible if they met all of the following criteria:

- involved children or adolescents aged 0–18 years after ischemic stroke;
- analyzed the timing of therapeutic intervention or its association with functional outcomes;
- concerned rehabilitation interventions, including physiotherapy, occupational therapy, speech and language therapy, neuropsychological intervention, or multidisciplinary management;
- were clinical studies, observational studies, systematic reviews, narrative reviews, clinical guidelines, or expert reports.

Sources were excluded if they:

- concerned hemorrhagic stroke without separate data for ischemic stroke;
- included exclusively adult populations;
- did not report therapeutic interventions or their timing;
- were editorials, comments, letters, or other publications without relevant clinical data.

### 2.4. Study Selection

Two reviewers independently screened all records in two stages. First, titles and abstracts were assessed for potential eligibility. Subsequently, full-text articles were evaluated against predefined inclusion and exclusion criteria. Before formal screening, the review team conducted a calibration exercise using a random sample of records to ensure consistent interpretation of eligibility criteria. Screening was performed independently by two reviewers for each record. Disagreements were resolved through discussion and, when necessary, consultation with a third reviewer until consensus was reached. Formal inter-rater agreement was not assessed, which may be considered a methodological limitation of the review process.

The initial search identified 742 records. After removing 71 duplicate records, 671 records remained for title and abstract screening. Following this stage, 447 records were excluded. A total of 224 full-text articles were assessed for eligibility. Of these, 203 publications were excluded for predefined reasons (Table S2), and 20 sources met the inclusion criteria and were included in the final review.

**Figure 1:**
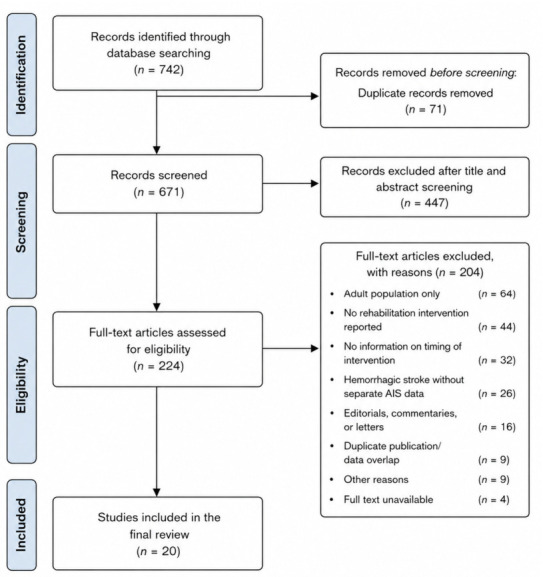
PRISMA flow diagram of study selection for the literature review. Note: The initial search was conducted on 31 January 2025 across PubMed, Scopus, Web of Science, Cochrane Library, and Google Scholar. Reasons for full-text exclusion are presented in Table S2.

The study selection process is presented in Figure 1.

### 2.5. Data Extraction and Synthesis

Data extraction was conducted using a standardized charting form developed by the review team. Extraction was performed independently by two reviewers and subsequently verified for accuracy and completeness. Any discrepancies were resolved through discussion and consensus. The following information was extracted from each included source: study design, population characteristics, stroke subtype, intervention time, type and intensity of rehabilitation, outcome measures, prognostic factors, and key findings relevant for early rehabilitation after pediatric ischemic stroke. Due to the substantial heterogeneity in study designs, populations, intervention characteristics, and outcome measures, the findings were synthesized narratively rather than quantitatively.

### 2.6. Quality Assessment

The methodological quality of the observational studies was evaluated using the Newcastle-Ottawa Scale (NOS). The studies were classified as high, moderate or low quality according to the domains of selection, comparability, and outcome assessment. Due to the limited number of empirical studies on pediatric ischemic stroke rehabilitation, the final synthesis included studies of different methodological designs, including clinical studies, reviews and guidelines. The methodological quality assessment of included observational studies is presented in Supplementary Table S1. The methodological quality assessment was conducted to provide additional context regarding the strength and limitations of the available evidence and was not used as a criterion for study exclusion. Quality ratings informed the interpretation of findings during narrative synthesis but did not determine study eligibility.

### 2.7. Operational Definition of Early Intervention

For the purposes of this review, the early therapeutic intervention was operationally classified according to the time from the onset of the stroke to rehabilitation initiation. These thresholds were developed by the review team based on a synthesis of adult stroke rehabilitation literature, in which early and ultra-early mobilization windows have been previously described, adapted taking into account available pediatric stroke rehabilitation evidence and the biological characteristics of the developing brain. As there is currently no universally accepted pediatric-specific classification of rehabilitation timing in the literature, the proposed categories represent author consensus informed by existing frameworks and should be interpreted accordingly. The classification is as follows:

- ultra-early: 0–7 days post-stroke;
- early: 7–30 days post-stroke;
- subacute early: 1–3 months post-stroke.

## 3. Results

A total of 20 evidence sources met the inclusion criteria. These included observational studies providing direct evidence regarding pediatric arterial ischemic stroke rehabilitation, pediatric stroke-specific reviews, clinical guidelines, expert consensus documents, and indirect evidence sources from related pediatric neurological conditions, including perinatal stroke, cerebral palsy, developmental neuroplasticity, and broader pediatric neurorehabilitation. Table 1 summarizes selected key evidence sources and indicates their type, population relevance, contribution to the review, and direct or indirect relevance to pediatric ischemic stroke rehabilitation.

### 3.1. Timing of Therapeutic Intervention

The available evidence suggests that earlier initiation of rehabilitation may be associated with improved functional outcomes following pediatric ischemic stroke. However, direct pediatric stroke evidence remains limited and is derived primarily from observational studies, while additional support originates from related pediatric neurological conditions and neuroplasticity research. Across the included sources, rehabilitation initiated during the acute or early subacute period following medical stabilization was generally associated with better functional recovery and greater independence. Conversely, delayed rehabilitation may increase reliance on compensatory movement strategies and reduce opportunities to optimize recovery. These findings support the hypothesis that an early post-stroke period may represent an important window for rehabilitation, although further prospective pediatric stroke studies are needed to confirm its clinical significance.

### 3.2. Types of Therapeutic Interventions

A wide range of therapeutic interventions was identified across the included evidence sources. However, the strength and specificity of evidence varied substantially between intervention types.

Direct pediatric ischemic stroke evidence primarily supported the use of multidisciplinary rehabilitation, including physiotherapy, occupational therapy, and speech and language therapy. These interventions were consistently described as core components of post-stroke management and were associated with improvements in motor function, functional independence, communication abilities, and participation in daily activities. Early initiation following medical stabilization was generally emphasized across pediatric stroke-specific studies and reviews [5,17,19].

Several additional rehabilitation approaches were supported predominantly by indirect evidence derived from related pediatric neurological populations. Constraint-induced movement therapy (CIMT), bimanual training, and intensive task-oriented upper-limb interventions demonstrated beneficial effects in children with unilateral cerebral palsy and perinatal stroke and may have relevance for selected children with pediatric ischemic stroke presenting with similar motor impairments [11,13]. However, direct pediatric ischemic stroke evidence for these interventions remains limited.

Similarly, family-centred rehabilitation models were supported primarily by studies involving cerebral palsy and broader pediatric neurorehabilitation populations. These studies suggested that active caregiver participation may improve therapy adherence, increase intervention intensity, and support long-term functional outcomes [14].

A detailed overview of standard and emerging therapeutic interventions, together with the corresponding evidence sources, is presented in Table 2A,B.

### 3.3. Neuroplasticity and Response to Therapy

The effectiveness of early intervention is closely related to the neuroplasticity of the developing brain. Experimental and clinical studies indicate that the early post-stroke period is characterized by increased susceptibility to neural reorganization, enabling the formation of new synaptic connections and functional reorganization of motor, cognitive, and language networks [18,21].

Adaptive neuroplasticity may facilitate recovery by promoting the recruitment of preserved neural pathways, strengthening functional networks, and supporting the restoration of motor and cognitive abilities. Early, intensive, and task-oriented rehabilitation is thought to enhance these adaptive processes by providing meaningful sensory and motor experiences during a period of heightened responsiveness to therapy [19,21]. However, neuroplasticity is not inherently beneficial. Maladaptive plasticity may occur when compensatory movement patterns, inefficient motor strategies, or non-use of the affected limb become reinforced through repetition. Such adaptations may initially improve task performance but can ultimately limit long-term recovery and functional potential. Consequently, the timing, specificity, and quality of therapeutic interventions are critical determinants of whether neural reorganization supports adaptive recovery or reinforces maladaptive behaviours.

The coexistence of developmental brain maturation and post-injury repair processes creates a unique therapeutic window in pediatric patients. This period may provide an opportunity to maximize adaptive neuroplasticity while minimizing the risk of maladaptive reorganization through early, individualized, and goal-directed rehabilitation [18,19,21].

### 3.4. Prognostic Factors

The reviewed evidence identified multiple factors that may influence the effectiveness of early rehabilitation following pediatric ischemic stroke. Age at stroke onset was consistently associated with recovery potential. Younger children may benefit from greater neuroplastic capacity; however, early brain injury may also result in delayed emergence of cognitive, behavioural, and language deficits as developmental demands increase over time [21].

Stroke characteristics constituted a major prognostic domain. Stroke subtype, lesion location, lesion extent, and lesion laterality were all associated with functional outcomes. Lesions involving the motor cortex, corticospinal tract, or language-related brain regions were generally associated with less favourable motor and communication outcomes. Left hemispheric lesions were more commonly associated with language impairment, whereas right hemispheric lesions were linked to visuospatial, attentional, and behavioural difficulties [18,19].

Several medical factors may additionally influence recovery trajectories. The presence of epilepsy or post-stroke seizures has been associated with poorer neurodevelopmental outcomes in some pediatric stroke populations. Similarly, underlying cardiac disease, one of the major risk factors for childhood arterial ischemic stroke, may contribute to greater clinical complexity and influence rehabilitation planning. The risk of recurrent stroke may further affect long-term outcomes and necessitate ongoing neurological monitoring and secondary prevention strategies [17,19].

Rehabilitation-related factors also appear important. Earlier initiation of therapy, greater rehabilitation intensity, and access to coordinated multidisciplinary services were generally associated with better functional outcomes. Conversely, delayed access to specialist pediatric rehabilitation may limit opportunities to optimize recovery during periods of heightened neuroplasticity [19,20,21].

Finally, environmental and socioeconomic factors may substantially influence rehabilitation success. Family engagement, caregiver capacity, socioeconomic circumstances, geographic access to specialist services, and continuity of rehabilitation support may affect treatment adherence, participation, and long-term developmental outcomes. Although these factors remain under-investigated in pediatric stroke populations, they are increasingly recognized as important determinants of recovery and healthcare equity [14].

Overall, prognosis after pediatric ischemic stroke is multifactorial and reflects the interaction between biological, clinical, rehabilitation-related, and environmental influences. Early individualized rehabilitation may help mitigate some adverse prognostic factors, although substantial heterogeneity in outcomes remains.

## 4. Discussion

The available evidence suggests that early therapeutic intervention may represent an important component of rehabilitation after pediatric ischemic stroke. Although direct pediatric stroke evidence remains limited and is derived primarily from observational studies, the included sources generally support consideration of rehabilitation initiation during the acute or early subacute period following medical stabilization. Earlier intervention may contribute to improved functional recovery and greater independence, although high-quality prospective pediatric stroke studies remain necessary to confirm these associations. The rationale for early intervention is grounded in the neuroplasticity of the developing brain. The early post-stroke period is thought to represent a critical window characterized by increased susceptibility to neural reorganization, potentially enabling more efficient adaptation to injury. Early, intensive, and task-oriented rehabilitation may support adaptive neuroplasticity and functional recovery, whereas delayed or poorly targeted interventions may increase the risk of reinforcing compensatory or maladaptive movement patterns. These mechanisms provide a biological rationale for age-specific rehabilitation strategies in pediatric populations.

An important finding of this review is that the available evidence supports a multidisciplinary rehabilitation approach. Direct pediatric stroke evidence primarily supports the use of physiotherapy, occupational therapy, speech and language therapy, and neuropsychological follow-up. Additional interventions, including constraint-induced movement therapy (CIMT), bimanual training, and family-centred rehabilitation approaches, are supported mainly by indirect evidence derived from unilateral cerebral palsy, perinatal stroke, and broader pediatric neurorehabilitation populations. Neuromodulation techniques remain experimental and currently have substantially less supporting evidence than CIMT or bimanual training.

**Figure 2:**
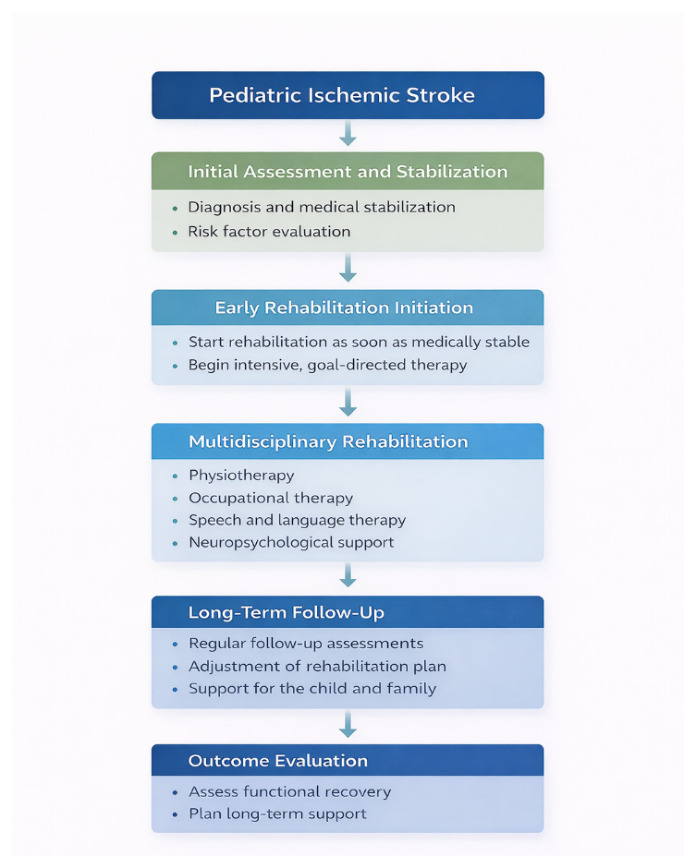
Proposed clinical pathway for early rehabilitation after pediatric ischemic stroke (own elaboration).

Based on the available evidence, Figure 2 presents an author-derived conceptual model of early rehabilitation after pediatric ischemic stroke. The pathway synthesizes findings identified in the present review and integrates the authors’ interpretation of current evidence. It should not be interpreted as a validated clinical protocol or formal clinical guideline. Rather, it is intended to support clinical discussion, future protocol development, and hypothesis generation.

From a clinical perspective, implementation of early rehabilitation remains challenging. Delayed recognition of pediatric stroke, limited availability of specialist pediatric stroke services, regional inequalities in access to rehabilitation, and difficulties during transition from inpatient treatment to outpatient and community-based care may all influence rehabilitation delivery and outcomes. Consequently, successful implementation of early rehabilitation requires not only evidence-based therapeutic strategies but also coordinated healthcare systems capable of providing timely diagnosis, specialist assessment, multidisciplinary treatment, and long-term follow-up.

### Limitations

This review has several limitations that should be considered when interpreting its findings.

First, no review protocol was prospectively registered. Although the review was conducted and reported according to the PRISMA-ScR recommendations, the absence of registration of the protocol can reduce transparency regarding methodological decisions made during the review process.

Second, the search was restricted to publications in English and Polish, which may have resulted in the exclusion of relevant studies published in other languages and introduced language bias.

Third, due to the limited availability of pediatric ischemic stroke rehabilitation studies, the evidence base was dominated by observational studies involving relatively small and heterogeneous patient populations. This limits the generalizability of the findings and precludes definitive conclusions regarding causal relationships between rehabilitation timing and functional outcomes. The absence of randomized controlled trials specifically examining rehabilitation timing and intensity in pediatric ischemic stroke remains a major gap in the literature.

Fourth, the review incorporated a wide range of evidence sources, including observational studies, systematic and narrative reviews, clinical guidelines, and expert reports. While this approach is consistent with scoping review methodology and was necessary to comprehensively map the available evidence, some conclusions rely on indirect evidence or expert consensus rather than pediatric stroke-specific empirical data.

Finally, the proposed clinical pathway presented in Figure 2 has not been externally validated and should be considered a conceptual framework based on the available evidence and the interpretation rather than as a validated clinical guideline or standard of care.

Despite these limitations, this review provides a comprehensive overview of current evidence regarding early rehabilitation after pediatric ischemic stroke and highlights important priorities for future research and clinical practice.

## 5. Conclusions and Research Directions

The findings of this scoping review suggest that early rehabilitation may be associated with improved functional outcomes in children after ischemic stroke; however, this interpretation is based on a limited, heterogeneous, and partly indirect body of evidence. The proposed benefits are supported primarily by observational pediatric stroke studies together with indirect evidence from related pediatric neurorehabilitation populations and neuroplasticity research. Early rehabilitation is biologically plausible because it coincides with a period of heightened neuroplasticity in the developing brain. However, evidence directly linking rehabilitation timing to neuroplastic mechanisms and long-term functional outcomes in pediatric ischemic stroke remains limited. The available literature suggests that multidisciplinary rehabilitation may help address motor, cognitive, communication, and participation-related needs, although the strength of evidence varies across intervention types and is frequently derived from indirect or observational sources.

The analyzed interventions were associated with potential benefits across several functional domains, although the quality and directness of the supporting evidence varied substantially between intervention types. Among specialized approaches, CIMT and bimanual therapy have the strongest evidence base for motor outcomes in children with unilateral brain injury. Neuromodulation has shown preliminary potential as an adjunct to conventional rehabilitation; however, its role remains investigational and requires further evaluation regarding safety, patient selection, stimulation parameters, and clinical efficacy.

Based on the available evidence, early, coordinated, and multidisciplinary rehabilitation is clinically plausible and may represent an important component of pediatric ischemic stroke care. Nevertheless, high-quality prospective studies and randomized controlled trials remain essential to establish standardized therapeutic protocols, define optimal timing and intensity parameters, and confirm long-term functional and neuropsychological benefits in this population. Access to early rehabilitation varies significantly between countries and institutions. Pediatric stroke units improve early detection and facilitate rapid intervention, but in many regions, delays in diagnosis and limited availability of specialized teams hinder optimal care.

Importantly, early rehabilitation should not be understood as universally intensive or protocol-driven; the risks of maladaptive neuroplasticity and therapy-related fatigue highlight the need for individualized, goal-directed approaches adapted to each child’s clinical status and developmental stage.

These findings underscore the necessity of cautious, individualized therapy to prevent maladaptive plasticity and the importance of further RCTs to strengthen the evidence. Clinically, the findings support consideration of an individualized, multidisciplinary approach to pediatric ischemic stroke rehabilitation while acknowledging the substantial limitations of the current evidence base. Future studies should incorporate standardized pediatric outcome measures, harmonized definitions of rehabilitation timing, minimum reporting standards for rehabilitation dose and intensity, long-term neuropsychological follow-up, and multicenter prospective registries.

## Figures and Tables

**Table 1:** Characteristics of selected key evidence sources relevant to early rehabilitation after pediatric ischemic stroke.

Reference	Source Type	Population/Evidence Source	Evidence Relevance	Main Contribution to the Review
Greenham et al., 2016 [5]	Cohort study	Children with arterial ischemic stroke	Direct evidence	Evaluated functional outcomes after pediatric arterial ischemic stroke and highlighted the potential benefits of earlier rehabilitation initiation.
Gordon et al., 2002 [6]	Observational study	Children with ischemic stroke	Direct evidence	Reported improvements in mobility and activities of daily living following rehabilitation interventions.
deVeber et al., 2000 [7]	Cohort study	Children with arterial ischemic stroke	Direct evidence	Identified prognostic factors influencing long-term outcomes and recovery after childhood stroke.
Pavlovic et al., 2006 [8]	Prospective follow-up study	Children with arterial ischemic stroke	Direct evidence	Assessed neuropsychological and functional outcomes two years after pediatric stroke.
Gordon et al., 2002 [6]	Review	Children with arterial ischemic stroke	Direct supportive evidence	Summarized current knowledge regarding rehabilitation, outcomes, and determinants of recovery after pediatric stroke.
Mastrangelo et al., 2022 [4]	Review	Pediatric ischemic stroke	Direct supportive evidence	Reviewed epidemiology, diagnosis, management, and rehabilitation considerations in childhood stroke.
Kirton, and deVeber, 2015 [9]	Narrative review	Pediatric arterial ischemic stroke and perinatal stroke	Direct supportive evidence	Discussed mechanisms of recovery, rehabilitation challenges, and future therapeutic directions.
Royal College of Physicians, 2017 [10]	Clinical guideline	Children and adolescents after stroke	Guideline/expert consensus	Recommended early multidisciplinary assessment, rehabilitation, and long-term follow-up.
Novak et al., 2017 [11]	Systematic review	Infants at risk of cerebral palsy, including perinatal brain injury populations	Indirect evidence—cerebral palsy/developmental neurological injury	Demonstrated the importance of early diagnosis and intervention during periods of heightened neuroplasticity; findings are not specific to pediatric ischemic stroke.
Kirton, 2017 [12]	Review	Children with perinatal stroke	Indirect evidence—perinatal stroke	Reviewed neuromodulation and rehabilitation approaches applicable to pediatric neurorehabilitation.
Sakzewski et al., 2014 [13]	Meta-analysis	Children with unilateral cerebral palsy	Indirect evidence—cerebral palsy	Demonstrated effectiveness of constraint-induced movement therapy and bimanual training for upper-limb function.
Dirks et al., 2011 [14]	Systematic review	Children with neurological disability and cerebral palsy	Indirect evidence—family-centred rehabilitation	Highlighted the importance of family participation and therapy intensity in rehabilitation outcomes.
Eyre, 2007 [15]	Review	Developing brain and pediatric neurorehabilitation	Indirect evidence—neuroplasticity	Provided biological rationale for early intervention based on developmental neuroplasticity.
Chen et al., 2002 [16]	Review	Stroke rehabilitation populations	Indirect evidence—general stroke rehabilitation	Discussed mechanisms of neural reorganization and recovery relevant to rehabilitation timing.
Liu et al. 2023 [17]	Neuroplasticity review	Experimental and clinical neuroplasticity research	Indirect evidence—neuroplasticity	Described critical windows of neuroplasticity and their implications for rehabilitation timing.
Hill et al. 2023 [18]	Review	Childhood stroke and developmental brain injury	Indirect evidence—developmental neuroscience	Examined the influence of age at injury and lesion characteristics on recovery trajectories.

Direct evidence refers to studies conducted in children with arterial ischemic stroke. Direct supportive evidence refers to reviews synthesizing pediatric stroke-specific literature. Indirect evidence refers to studies of related pediatric neurological conditions (e.g., perinatal stroke, cerebral palsy, unilateral brain injury), neuroplasticity research, general stroke rehabilitation literature, clinical guidelines, and expert consensus documents used to provide contextual support where pediatric stroke-specific evidence was limited.

**Table 2:** (**A**) Common rehabilitation approaches in early rehabilitation after pediatric stroke. (**B**) Emerging or specialized rehabilitation approaches relevant to pediatric stroke rehabilitation.

**(A)**					
**Intervention**	**Main Therapeutic Target**	**Typical Techniques**	**Evidence Sources**	**Evidence Relevance**	**Reported Benefits**
Physical therapy	Gross motor function, postural control, balance, and gait	Neurodevelopmental therapy, functional training, strengthening, balance and gait training	Greenham et al. [5]; Gordon et al. [6]; Ganesan [20]	Direct pediatric stroke evidence/direct supportive evidence	Improved mobility, postural control, gait, and functional independence
Occupational therapy	Fine motor function, upper-limb use, independence in activities of daily living	Task-oriented training, activities of daily living training, sensory-motor strategies, environmental adaptation	Greenham et al. [5]; Gordon et al. [6]; Ganesan [20]	Direct pediatric stroke evidence/direct supportive evidence	Improved manual skills, daily functioning, participation, and independence
Speech and language therapy	Communication, language development, swallowing, and cognitive-communication skills	Language therapy, articulation training, communication support, swallowing assessment and therapy when indicated	Pavlovic et al. [8]; Ganesan [20]	Direct pediatric stroke evidence/direct supportive evidence	Improved communication outcomes and support for developmental participation
Neuropsychological support	Cognitive, behavioural, emotional, and educational functioning	Neuropsychological assessment, cognitive rehabilitation, school reintegration support, behavioural strategies	Pavlovic et al. [8]; Greenham et al. [5]	Direct pediatric stroke evidence	Identification and management of long-term cognitive and behavioural sequelae
Family-centred rehabilitation	Therapy adherence, home practice, participation, and long-term support	Parent education, home programmes, caregiver coaching, goal-setting with the family	Dirks et al. [14]	Indirect evidence—pediatric neurorehabilitation/cerebral palsy	Increased therapy engagement, improved continuity of care, and enhanced support for long-term outcomes
Multidisciplinary rehabilitation	Comprehensive functional recovery across motor, cognitive, communication, and participation domains	Coordinated physical therapy, occupational therapy, speech and language therapy, neuropsychological support, and follow-up care	Royal College of Physicians [10]; Ganesan [20]; Greenham et al. [5]	Guideline/expert consensus and direct supportive evidence	More coordinated care, broader functional assessment, and improved continuity of rehabilitation
**(B)**					
**Intervention**	**Main Therapeutic Target**	**Typical Techniques**	**Evidence Sources**	**Evidence Relevance**	**Current Evidence Summary**
Constraint-induced movement therapy (CIMT)	Functional use of the affected upper limb	Constraint of the less-affected limb combined with intensive task-oriented practice of the affected limb	Sakzewski et al. [13]; Novak et al. [12]	Indirect evidence—unilateral cerebral palsy/pediatric neurological injury	Evidence supports benefit in children with unilateral motor impairment, particularly unilateral cerebral palsy. Direct pediatric ischemic stroke evidence remains limited.
Bimanual training	Bilateral coordination and functional use of both hands	Structured bilateral task practice, goal-directed hand-use activities	Sakzewski et al. [13]	Indirect evidence—unilateral cerebral palsy	May improve functional hand use and bilateral coordination in children with unilateral motor impairment; pediatric stroke-specific evidence remains limited.
Early intensive motor stimulation	Motor development and activity-dependent neuroplasticity	Early positioning, guided motor practice, task-specific stimulation, enriched developmental activities	Novak et al. [11]; Eyre [15]	Indirect evidence—developmental neurorehabilitation/neuroplasticity	Supports the biological rationale for early intervention during periods of heightened neuroplasticity; not specific to pediatric ischemic stroke.
Neuromodulation	Cortical excitability and motor network reorganization	Transcranial magnetic stimulation, transcranial direct current stimulation combined with rehabilitation	Kirton [12]	Indirect evidence—perinatal stroke/experimental pediatric neurorehabilitation	Preliminary evidence suggests potential benefit in selected pediatric populations. Safety, patient selection, stimulation parameters, and clinical efficacy require further investigation before routine clinical use.
Intensive task-oriented upper-limb therapy	Upper-limb function and participation	Repetitive goal-directed practice, functional task training, structured home practice	Sakzewski et al. [13]; Novak et al. [12]	Indirect evidence—unilateral cerebral palsy/pediatric neurological injury	May be relevant for children with pediatric ischemic stroke and unilateral motor deficits, but direct evidence remains limited.

Note: Direct pediatric stroke evidence refers to studies conducted in children with arterial or ischemic stroke. Direct supportive evidence refers to pediatric stroke-specific reviews. Indirect evidence refers to studies from related pediatric neurological populations, including perinatal stroke, unilateral cerebral palsy, broader pediatric neurorehabilitation, and neuroplasticity research. Guideline/expert consensus refers to recommendations derived from clinical guidelines or expert documents rather than primary pediatric ischemic stroke rehabilitation trials. Abbreviations: CIMT, constraint-induced movement therapy.

## Data Availability

No new empirical data were generated in this study. The data supporting the findings of this review were extracted from published literature and are available within the cited articles and the Supplementary Materials accompanying this manuscript.

## Supplementary Materials

The following supporting information can be downloaded at: https://www.mdpi.com/article/10.3390/children13070866/s1, Table S1. Methodological quality assessment of included observational studies using the Newcastle–Ottawa Scale (NOS); Table S2. Reasons for full-text exclusion; Table S3. Full search strategies used in each database.
