# Supplementary material for: Early Rehabilitation in Children After Ischemic Stroke—Importance and Effects: A Scoping Review
Source: Children (Basel). 2026 Jun 29;13(7):866. doi: 10.3390/children13070866 (PMC13406796; doi:10.3390/children13070866)
Supplement: Supplementary file 1 [file children-13-00866-s001.zip › children-4355002-supplementary.pdf]

Supplementary Table S1. Methodological quality assessment of included observational studies using the Newcastle–Ottawa Scale (NOS).

Study	Study design	Selection	Comparability	Outcome	Total NOS score	Quality rating
Greenham et al., 2016	Cohort study	4/4	1/2	3/3	8/9	High
Gordon et al., 2002	Observational study	3/4	1/2	2/3	6/9	Moderate
deVeber et al., 2000	Cohort study	4/4	1/2	2/3	7/9	High
Pavlovic et al., 2006	Observational follow-up study	3/4	1/2	3/3	7/9	High

Scoring criteria: High quality = 7–9 points; moderate quality = 4–6 points; low quality = 0–3 points.

Abbreviations: NOS, Newcastle–Ottawa Scale.

Note: The NOS assessment was applied only to observational studies. Reviews, systematic reviews, meta-analyses, clinical guidelines, and expert consensus documents were not assessed using NOS because this tool is designed for non-randomized observational studies.

Supplementary Table S2. Reasons for full-text exclusion

Reason for exclusion	n
Adult population only	64
Hemorrhagic stroke without separate AIS data	26
No rehabilitation intervention reported	44
No information on timing of intervention	32
Editorials, commentaries, letters, or conference abstracts	16
Duplicate publication or overlapping dataset	9
Full text unavailable	4
Other predefined exclusion criteria	8
Total	203

Supplementary Table S3. Full search strategies used in each database

Database	Search strategy
**PubMed/MEDLINE**	("Stroke"[Mesh] OR "Brain Ischemia"[Mesh] OR "arterial ischemic stroke" OR "ischemic stroke" OR "childhood stroke" OR "pediatric stroke" OR "paediatric stroke" OR "perinatal stroke") AND ("Rehabilitation"[Mesh] OR rehabilitation OR neurorehabilitation OR "early intervention" OR "early rehabilitation" OR physiotherapy OR "physical therapy" OR "occupational therapy" OR "speech therapy" OR "speech and language therapy" OR "constraint-induced movement therapy" OR CIMT OR "bimanual therapy" OR neuromodulation) AND (child* OR pediatric OR paediatric OR infant* OR adolescent*) AND ("functional outcome*" OR recovery OR neuroplasticity OR hemiparesis OR "motor outcome*" OR "upper limb function" OR disability OR participation)
**Scopus**	TITLE-ABS-KEY (("arterial ischemic stroke" OR "ischemic stroke" OR "childhood stroke" OR "pediatric stroke" OR "paediatric stroke" OR "perinatal stroke") AND (rehabilitation OR neurorehabilitation OR "early intervention" OR physiotherapy OR "physical therapy" OR "occupational therapy" OR "speech therapy" OR "constraint-induced movement therapy" OR CIMT OR "bimanual therapy" OR neuromodulation) AND (child* OR pediatric OR paediatric OR infant* OR adolescent*) AND ("functional outcome*" OR recovery OR neuroplasticity OR hemiparesis OR "motor outcome*" OR "upper limb function"))
**Web of Science Core Collection**	TS=(("arterial ischemic stroke" OR "ischemic stroke" OR "childhood stroke" OR "pediatric stroke" OR "paediatric stroke" OR "perinatal stroke") AND (rehabilitation OR neurorehabilitation OR "early intervention" OR physiotherapy OR "physical therapy" OR "occupational therapy" OR "speech therapy" OR "constraint-induced movement therapy" OR CIMT OR "bimanual therapy" OR neuromodulation) AND (child* OR pediatric OR paediatric OR infant* OR adolescent*) AND ("functional outcome*" OR recovery OR neuroplasticity OR hemiparesis OR "motor outcome*" OR "upper limb function"))
**Cochrane Library**	("arterial ischemic stroke" OR "ischemic stroke" OR "childhood stroke" OR "pediatric stroke" OR "perinatal stroke") AND (rehabilitation OR neurorehabilitation OR "early intervention" OR physiotherapy OR "occupational therapy" OR "speech therapy" OR "constraint-induced movement therapy" OR CIMT OR neuromodulation) AND (child* OR pediatric OR paediatric OR infant* OR adolescent*)
**Google Scholar**	All of the following terms were used in different combinations: "pediatric stroke", "childhood stroke", "arterial ischemic stroke", "early rehabilitation", "early intervention", neurorehabilitation, physiotherapy, occupational therapy, speech therapy, hemiparesis, "motor outcome", recovery, neuroplasticity, "constraint-induced movement therapy", CIMT, "upper limb therapy", and bimanual therapy. The first 330 records sorted by relevance were screened.

Limits applied to all databases: publication years 2000–January 2025; English or Polish language; studies involving children and adolescents (0–18 years).

Abbreviations: CIMT, constraint-induced movement therapy.
